# Triple-Dose Furmonertinib for Leptomeningeal Metastases in Advanced Epidermal Growth Factor Receptor (EGFR) L858R-Mutated Lung Adenocarcinoma: A Case Report

**DOI:** 10.7759/cureus.104428

**Published:** 2026-02-28

**Authors:** Hong Wu, Lei Li, Jing Yang, Yuan Tian, Hui Wang

**Affiliations:** 1 Department of Radiation Oncology, Affiliated Hospital of Shandong University of Traditional Chinese Medicine, Jinan, CHN; 2 Department of Oncology, Binzhou Medical University Hospital, Binzhou, CHN; 3 Orthopedic Disease Center, Affiliated Hospital of Shandong University of Traditional Chinese Medicine, Jinan, CHN

**Keywords:** cns metastases, egfr mutation, furmonertinib, leptomeningeal metastases, nsclc

## Abstract

Leptomeningeal metastases (LM) represent a severe and life-threatening manifestation of advanced non-small cell lung cancer (NSCLC). Despite advances in epidermal growth factor receptor (EGFR)-targeted therapies, central nervous system involvement continues to present major therapeutic challenges. We report a 73-year-old woman with EGFR L858R-mutated NSCLC who developed LM after multiple lines of therapy, including gefitinib, osimertinib, chemotherapy, anti-angiogenic therapy, and radiotherapy. Treatment with high-dose furmonertinib (240 mg daily) combined with bevacizumab resulted in symptom relief and additional survival. Remarkably, her overall survival exceeded six years from initial diagnosis. This case highlights the potential role of dose-escalated furmonertinib as salvage therapy in LM after osimertinib resistance and underscores the importance of sequential and multimodal management in advanced EGFR-mutant NSCLC.

## Introduction

Leptomeningeal metastases (LM) represent one of the most challenging forms of central nervous system (CNS) progression in lung cancer. Non-small cell lung cancer (NSCLC) patients with epidermal growth factor receptor (EGFR) mutations are at higher risk of LM compared to EGFR wild-type cases, with an incidence approaching 9% [1,2]. In clinical practice, LM is typically diagnosed based on characteristic findings on contrast-enhanced magnetic resonance imaging (MRI), such as leptomeningeal enhancement, while cerebrospinal fluid (CSF) cytology serves as a supportive or confirmatory diagnostic tool. Historically, LM was associated with a median survival of less than one year, even with intrathecal chemotherapy, whole-brain radiotherapy (WBRT), or systemic chemotherapy [3].

Third-generation EGFR tyrosine kinase inhibitors (TKIs), especially osimertinib, have improved CNS control. However, LM progression remains a critical barrier, with limited effective treatment options after osimertinib resistance [4,5]. Furmonertinib, a third-generation EGFR-TKI with favorable CNS penetration, has shown encouraging efficacy in both clinical trials and real-world studies, particularly at higher doses [6]. A daily dose of 240 mg is commonly referred to as a “triple-dose” regimen, corresponding to three times the standard recommended dose of 80 mg. Nevertheless, detailed case reports describing its use in EGFR L858R-mutated NSCLC with LM remain scarce.

We present a patient with EGFR L858R-mutated lung adenocarcinoma who survived more than six years after initial diagnosis, including additional survival following LM managed with high-dose furmonertinib and bevacizumab.

## Case presentation

A 73-year-old nonsmoking woman was admitted to the thoracic surgery department in October 2018 with complaints of chest and back pain. She underwent video-assisted thoracoscopic left upper lobectomy with mediastinal lymph node dissection. Intraoperatively, a 5 × 4 × 4 cm mass with ill-defined margins and visceral pleural indentation was observed in the left upper lobe. Postoperative pathology confirmed invasive adenocarcinoma with visceral pleural invasion, and one of seven lymph nodes was positive for metastasis. Molecular testing revealed an EGFR exon 21 L858R mutation, and the patient was diagnosed with left lung adenocarcinoma, staged as T2bN1M0 (stage IIB) according to the American Joint Committee on Cancer (AJCC) 8th edition TNM classification [7].

Following surgery, the patient received adjuvant gefitinib starting in November 2018. She remained on gefitinib until January 2022, during which regular follow-up demonstrated no evidence of recurrence or metastasis.

**Figure 1:**
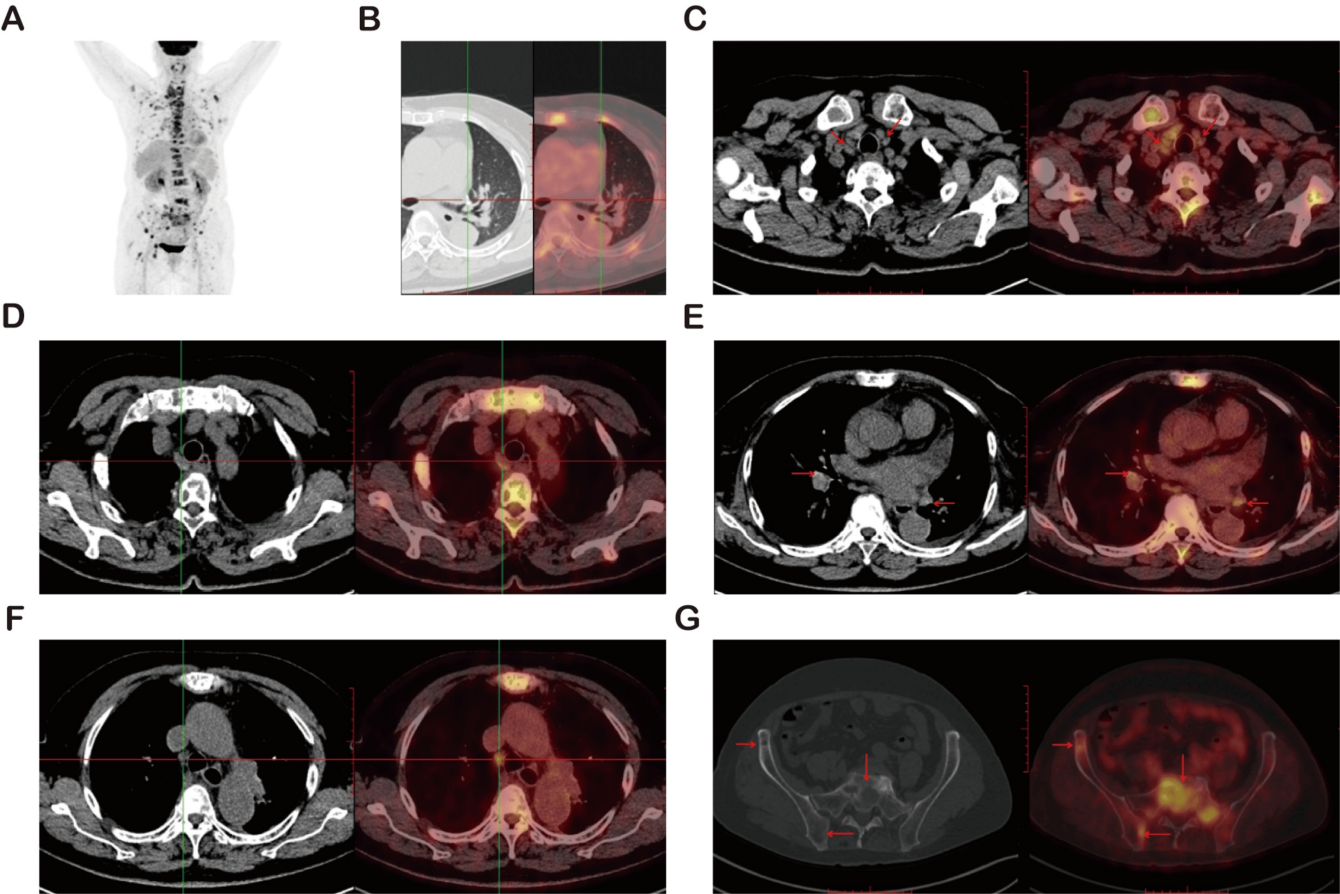
PET-CT Findings Suggestive of Widespread Lymphatic and Skeletal Metastases (A) PET-CT demonstrates multiple hypermetabolic lesions involving lymph nodes and bones throughout the body. (B) No abnormal FDG uptake detected at the previous surgical site. (C) Increased FDG uptake in right paratracheal lymph nodes (SUVmax 4.3). (D) Hypermetabolic activity in upper paratracheal lymph nodes (SUVmax 3.8). (E) Bilateral hilar lymph nodes with elevated FDG uptake (SUVmax 4.5). (F) Marked uptake in lower paratracheal lymph nodes (SUVmax 6.2). (G) High FDG uptake in the sacrum, indicative of osseous metastasis (SUVmax 7.2).

In October 2023, she presented again with recurrent chest and back pain. PET-CT revealed mediastinal and hilar lymphadenopathy together with widespread skeletal metastases, indicating postoperative recurrence (Figure 1).

**Figure 2:**
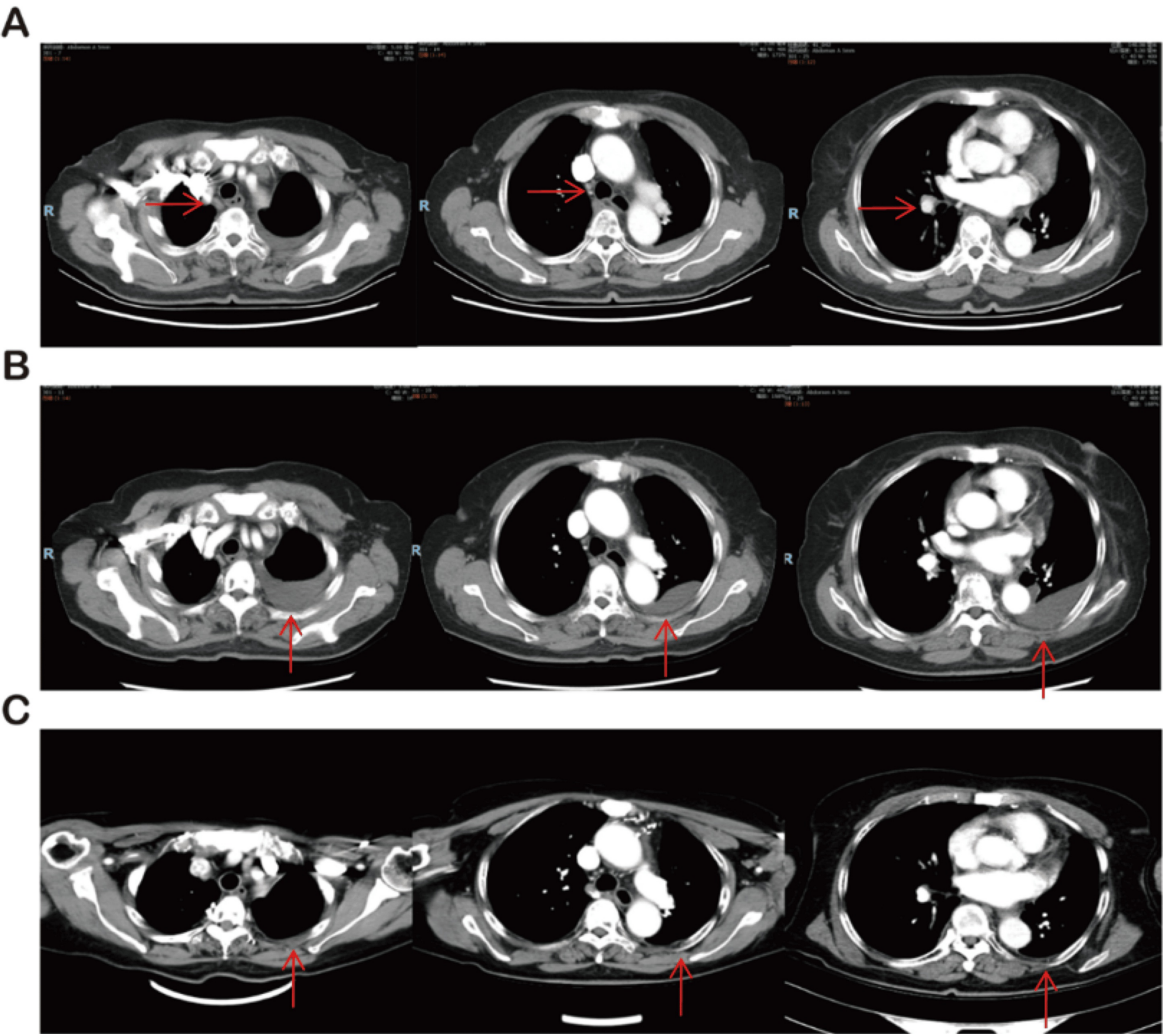
Serial CT imaging illustrating the evolution of mediastinal lymphadenopathy and pleural effusion. (A) Chest CT on December 29, 2023, after two months of osimertinib treatment, shows a reduction in mediastinal lymph node size. (B) On March 1, 2024, malignant pleural effusion was identified, prompting initiation of bevacizumab combined with chemotherapy. (C) On December 16, 2024, follow-up CT revealed improved mediastinal lymphadenopathy and reduced pleural effusion after treatment.

She was started on osimertinib 80 mg daily, administered from October 2023 to March 2024, along with palliative radiotherapy to the T2-T3 vertebrae (30 Gy in 10 fractions). Follow-up CT scans showed partial regression of nodal disease, consistent with a partial response by RECIST (Response Evaluation Criteria in Solid Tumors) criteria, but progressive left pleural effusion developed (Figure 2). The patient experienced chest tightness and dyspnea and underwent thoracentesis, followed by intrapleural chemotherapy to control malignant effusion.

From March to December 2024, she received a total of 12 cycles of pemetrexed- and cisplatin-based chemotherapy combined with bevacizumab, followed by maintenance therapy. Bevacizumab was administered every 21 days at a weight-based dose. Imaging demonstrated stable disease in the mediastinum and hilum and a reduction in pleural effusion compared with previous assessments (Figure 2).

**Figure 3:**
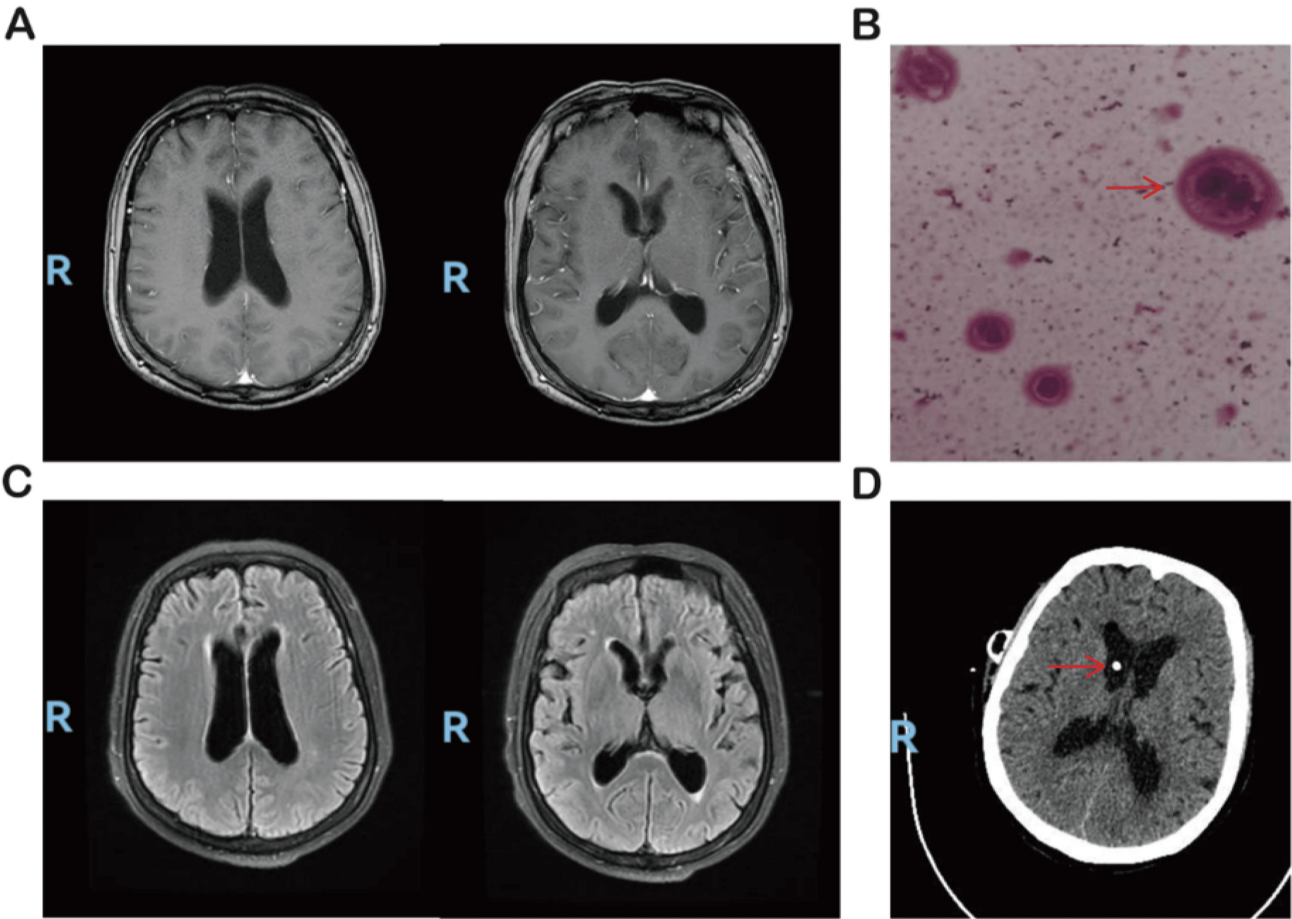
Neuroimaging and cerebrospinal fluid findings associated with leptomeningeal metastases. (A) Brain MRI on January 1, 2025, revealed no significant abnormalities. (B) Cerebrospinal fluid cytology showed large atypical cells with abundant cytoplasm and eccentrically located, hyperchromatic nuclei. (C) After initiating furmonertinib 240 mg daily, the patient’s dizziness improved; follow-up MRI on April 14, 2025, remained unremarkable. (D) On May 19, 2025, cerebrospinal fluid diversion was performed to relieve intracranial hypertension; high-density shadow in the ventricle represents the drainage catheter.

In January 2025, the patient developed new neurological symptoms, including dizziness and headache. Cranial MRI was unremarkable. Lumbar puncture revealed an elevated opening pressure of 26 cmH2O, and cerebrospinal fluid cytology was positive for malignant adenocarcinoma cells, consistent with leptomeningeal metastasis (Figure 3). Cerebrospinal fluid-based molecular testing for resistance mechanisms was not performed. She was therefore started on high-dose furmonertinib (240 mg daily) in combination with bevacizumab, resulting in progressive relief of neurological symptoms within the first several weeks after treatment initiation. No long-term corticosteroids or opioid analgesics were required during the initial neurological response.

During treatment with high-dose furmonertinib, the patient experienced decreased appetite and fatigue. No grade ≥3 treatment-related adverse events were observed, and the treatment was generally well tolerated.

In May 2025, she experienced recurrent severe headache and vomiting, and a cerebrospinal fluid diversion procedure was performed to relieve intracranial hypertension (Figure 3).

Despite initial improvement, she developed persistent high fever and delirium in July 2025 and eventually died of presumed intracranial infection.

**Figure 4:**
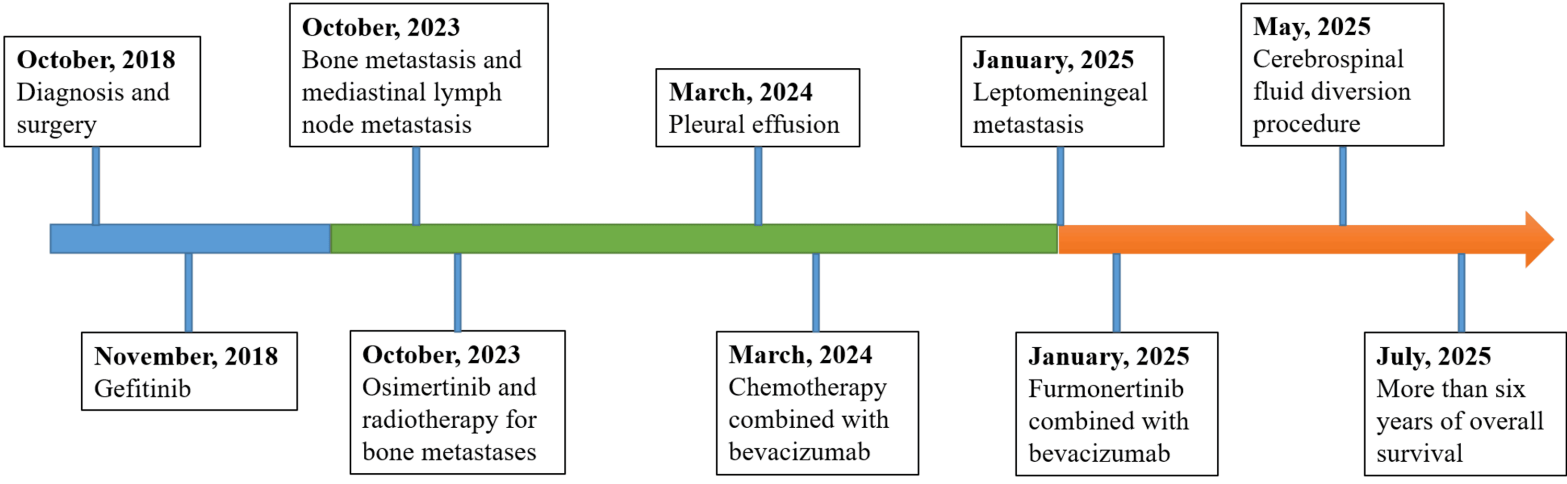
Chronological timeline of the patient’s clinical course, treatment interventions, and key clinical events.

A chronological overview of the disease trajectory, treatment interventions, and outcomes is summarized in Figure 4, highlighting the sequential application of surgery, targeted therapies, chemotherapy, radiotherapy, and neurosurgical procedures, as well as the dynamic adaptation of strategies in response to evolving disease progression.

## Discussion

LM are a devastating complication of NSCLC, often resulting in severe neurological symptoms, limited therapeutic options, and shortened survival [8]. Patients with EGFR-mutant NSCLC are particularly vulnerable, with an incidence of LM approaching 9%, compared to 3%-5% in the general NSCLC population [9]. Historically, prognosis has been poor, with a median overall survival (OS) of only 3-11 months despite intrathecal chemotherapy, whole-brain radiotherapy, or systemic chemotherapy [10]. The development of third-generation EGFR tyrosine kinase inhibitors (TKIs), which can penetrate the blood-brain barrier, has significantly improved CNS outcomes, yet LM continues to represent a major clinical challenge [11].

Furmonertinib is a novel, third-generation EGFR-TKI designed to achieve higher CNS drug exposure. Preclinical studies have demonstrated that its concentrations in brain tissue substantially exceed those in plasma [12]. Clinical evidence has confirmed these findings. In the pivotal phase III FURLONG trial, furmonertinib showed superior CNS progression-free survival (20.8 vs. 9.8 months) and higher CNS response rates compared with gefitinib [11]. Real-world studies have further supported its CNS activity, particularly at higher doses. In one prospective study of 48 patients with LM, high-dose furmonertinib (240 mg daily) achieved a CNS disease control rate of 92% and a median OS of 8.4 months [6]. Several case reports also support dose escalation after osimertinib failure, with rapid neurological improvement and survival benefits [13,14]. These findings highlight the potential of furmonertinib as a salvage therapy for LM.

The present case provides several important insights. First, our patient achieved an OS exceeding six years from initial diagnosis, far longer than typically reported in stage IIB EGFR-mutant NSCLC with LM. This outcome underscores the value of sequential, individualized treatment strategies incorporating surgery, adjuvant EGFR-TKI, systemic chemotherapy, anti-angiogenic therapy, radiotherapy, and finally high-dose furmonertinib. Second, after failure of osimertinib and multiple systemic therapies, the initiation of furmonertinib 240 mg daily combined with bevacizumab provided meaningful neurological improvement and extended survival. This clinical benefit aligns with emerging evidence suggesting that high-dose furmonertinib can achieve therapeutic CNS concentrations and overcome some mechanisms of resistance. Third, the case illustrates the potential utility of combining furmonertinib with anti-angiogenic therapy, which may further enhance CNS disease control, as suggested in recent retrospective analyses [15,16].

## Conclusions

This report highlights a rare clinical course of EGFR L858R-mutant non-small cell lung cancer characterized by late-onset LM after multiple lines of therapy. LM remain a critical therapeutic challenge in EGFR-mutant disease, often developing despite prior tyrosine kinase inhibitor treatment. The patient’s favorable response suggests that high-dose furmonertinib (240 mg daily), particularly in combination with anti-angiogenic therapy, was associated with meaningful neurological symptom control in this clinical context, even after osimertinib failure. It should be noted that the observed clinical course occurred in the setting of multiple therapeutic interventions, including prior systemic treatments and cerebrospinal fluid diversion, which may have contributed to symptom improvement. Comprehensive and individualized sequencing of multimodal therapies appears crucial for sustained disease control, and dose-escalation strategies integrated within multidisciplinary management may represent a potential option for selected patients. Future prospective studies are warranted to further define the optimal role of high-dose furmonertinib in this difficult-to-treat population.

## References

[1] Ozcan G, Singh M, Vredenburgh JJ (2023). Leptomeningeal metastasis from non-small cell lung cancer and current landscape of treatments. Clin Cancer Res.

[2] Bortolot M, Huijs JW, Brandsma D, Compter A, van Geel RM, Hendriks LE (2025). Advancing leptomeningeal metastases treatment in EGFR-mutated non-small cell lung cancer: lessons from the BLOSSOM trial. Transl Lung Cancer Res.

[3] Jia C, Xu Q, Zhao L, Kong F, Jia Y (2024). Therapeutic role of EGFR - tyrosine kinase inhibitors in non-small cell lung cancer with leptomeningeal metastasis. Transl Oncol.

[4] Batra U, Biswas B, Prabhash K, Krishna MV (2023). Differential clinicopathological features, treatments and outcomes in patients with Exon 19 deletion and Exon 21 L858R EGFR mutation-positive adenocarcinoma non-small-cell lung cancer. BMJ Open Respir Res.

[5] Park S, Baldry R, Jung HA (2024). Phase II efficacy and safety of 80 mg osimertinib in patients with leptomeningeal metastases associated with epidermal growth factor receptor mutation-positive non-small cell lung cancer (Blossom). J Clin Oncol.

[6] Chen H, Yang S, Wang L (2025). High-dose Furmonertinib in patients with EGFR-mutated NSCLC and leptomeningeal metastases: a prospective real-world study. J Thorac Oncol.

[7] Amin MB, Greene FL, Edge SB (2017). The eighth edition AJCC cancer staging manual: continuing to build a bridge from a population-based to a more “personalized” approach to cancer staging. CA Cancer J Clin.

[8] Lin G, Wang Y, Xin T (2025). Chinese expert consensus on leptomeningeal metastases of lung cancer. Thorac Cancer.

[9] Malhotra J, Mambetsariev I, Gilmore G (2025). Targeting CNS metastases in non-small cell lung cancer with evolving approaches using molecular markers: a review. JAMA Oncol.

[10] Wang Y, Yang X, Li NJ, Xue JX (2022). Leptomeningeal metastases in non-small cell lung cancer: diagnosis and treatment. Lung Cancer.

[11] Li Z, Lu S (2023). Third-generation EGFR tyrosine kinase inhibitor for central nervous system metastases EGFR-mutant NSCLC: current evidence and future perspectives. J Thorac Oncol.

[12] Shi Y, Chen G, Wang X (2022). Central nervous system efficacy of Furmonertinib (Ast2818) versus gefitinib as first-line treatment for EGFR-mutated NSCLC: results from the furlong study. J Thorac Oncol.

[13] Jia G, Bashir S, Ye M, Li Y, Lai M, Cai L, Xu M (2024). Furmonertinib and intrathecal pemetrexed chemotherapy rechallenges osimertinib-refractory leptomeningeal metastasis in a non-small cell lung cancer patient harboring EGFR20 R776S, C797S, and EGFR21 L858R compound EGFR mutations: a case report. Anticancer Drugs.

[14] Chen T, Chen J, Liu DS (2023). Successful therapy using high-dose furmonertinib for non-small cell lung cancer with leptomeningeal metastasis: a case report and literature review. Front Oncol.

[15] Qi R, Fu X, Yu Y, Xu H, Shen M, He S, Lv D (2023). Efficacy and safety of re-challenging 160 mg furmonertinib for advanced NSCLC after resistance to third-generation EGFR-TKIs targeted agents: a real-world study. Lung Cancer.

[16] Xu Z, Hao X, Wang Q, Yang K, Li J, Xing P (2023). Intracranial efficacy and safety of furmonertinib 160 mg with or without anti-angiogenic agent in advanced NSCLC patients with BM/LM as salvage therapy. BMC Cancer.

